# Replicating Viral Vector-Based Vaccines for COVID-19: Potential Avenue in Vaccination Arena

**DOI:** 10.3390/v14040759

**Published:** 2022-04-06

**Authors:** Vivek P. Chavda, Rajashri Bezbaruah, Mansi Athalye, Palak K. Parikh, Abu Sufiyan Chhipa, Snehal Patel, Vasso Apostolopoulos

**Affiliations:** 1 Department of Pharmaceutics and Pharmaceutical Technology, L. M. College of Pharmacy, Ahmedabad 380009, Gujarat, India; mansi.athalye@lmcp.ac.in; 2 Department of Pharmaceutics, K B Institute of Pharmaceutical Education and Research, Kadi Sarva Vishwavidhyalaya, Gandhinagar 382023, Gujarat, India; 3 Department of Pharmaceutical Sciences, Faculty of Science and Engineering, Dibrugarh University, Dibrugarh 786004, Assam, India; rajashribezbaruah@dibru.ac.in; 4 Department of Pharmaceutical Chemistry and Quality Assurance, L. M. College of Pharmacy, Ahmedabad 380009, Gujarat, India; palak.parikh@lmcp.ac.in; 5 Department of Pharmacology, Institute of Pharmacy, Nirma University, Ahmedabad 382481, Gujarat, India; 20ftphdp63@nirmauni.ac.in (A.S.C.); snehal.patel@nirmauni.ac.in (S.P.); 6 Institute for Health and Sport, Victoria University, Melbourne, VIC 3030, Australia

**Keywords:** vaccine, SARS-CoV-2, viral vector-based vaccine, replicating viral vector, COVID-19, vaccine efficacy

## Abstract

The “severe acute respiratory syndrome coronavirus 2 (SARS-CoV-2)” is the third member of human coronavirus (CoV) that is held accountable for the current “coronavirus disease 2019 (COVID-19)” pandemic. In the past two decades, the world has witnessed the emergence of two other similar CoVs, namely SARS-CoV in 2002 and MERS-CoV in 2013. The extent of spread of these earlier versions was relatively low in comparison to SARS-CoV-2. Despite having numerous reports inclined towards the zoonotic origin of the virus, one cannot simply sideline the fact that no animal originated CoV is thus far identified that is considered similar to the initial edition of SARS-CoV-2; however, under-sampling of the diverse variety of coronaviruses remains a concern. Vaccines are proved to be an effective tool for bringing the end to such a devastating pandemic. Many vaccine platforms are explored for the same but in this review paper, we will discuss the potential of replicating viral vectors as vaccine carriers for SARS-CoV-2.

## 1. Introduction

A vaccine belongs to the class of biological products that use antigenic substances to safely induce an immune response, thus creating antibodies that confer protection against targeted infections by strengthening the immune defense [1,2,3]. The era of vaccination was created two centuries ago in 1796 by Edward Jenner’s scientific investigations on the prevention of smallpox, through inoculation with the cowpox virus [4]. Different vaccines have been developed in the late 19th and early 20th centuries which have considerably reduced the burden of infectious diseases caused by bacteria and viruses [5,6]. Traditionally, vaccines have been categorized as live attenuated and whole killed organism or inactivated bacterial toxins. In addition to the whole-cell based approach of live attenuated or inactivated vaccines, several other types of vaccine have been developed including viral vectors, subunit vaccines (e.g., recombinant peptide/protein, conjugate, or polysaccharide), virus-like particles, nucleic acid-based DNA and RNA vaccines, etc. [2,7,8,9,10]. Since the emergence of the “severe acute respiratory syndrome coronavirus 2 (SARS-CoV-2)” pandemic, different strategies have been used to fight against the virus including social distancing, masks, drug repurposing, convalescent plasma (CP) therapy, novel antiviral agents, therapeutic antibodies, and vaccines [11,12,13]. Considering the devastating impact of “coronavirus disease 2019 (COVID-19)”, vaccination is one of the most promising clinical measures to decrease mortality and suppress the pandemic [14]. According to the World Health Organization (WHO) COVID-19 vaccine tracker and landscape, around 140 vaccines are under clinical development and 194 vaccines are under pre-clinical development as of March 2022 [15]. Currently, 10 vaccine candidates have been approved under emergency use by authorization of WHO, which have been developed by four different vaccine platforms including non-replicating viral vaccine, mRNA-based vaccine, protein subunit viral vaccine, and inactivated whole virus vaccine [16]. Pfizer/BioNTech COVID-19 vaccine (Comirnaty^®^) is the first COVID-19 vaccine fully approved by the U.S. Food and Drug Administration (USFDA) [17]. The efficient development, dissemination, and deployment of COVID-19 vaccines is a noteworthy science success story and currently approved vaccines have been found effective in the prevention of COVID-19, specifically against the severe diseased state [18]. Since late 2020, different genetic SARS-CoV-2 variants have emerged and circulated throughout the pandemic [19]. Despite the availability of different COVID-19 vaccines, there are still significant challenges in the context of recent pandemic waves. These challenges include the emergence of new SARS-CoV-2 variants and the effectiveness of COVID-19 vaccines against them, the effectiveness of vaccines in reducing the transmission of SARS-CoV-2, achieving herd immunity to combat the pandemic, as well as uncertainty about the duration of vaccine protection [20,21]. The rapid emergence of SARS-CoV-2 variants of concern (VOCs), such as Delta (B.1.617.2) and Omicron (B.1.1.529), is believed to result from their higher transmissibility and their ability to evade immunity conferred from vaccination or past infections [20,22]. As a comprehensive understanding of SARS-CoV-2 and its variants is still evolving, the outcomes for COVID-19 vaccines are required to be evaluated critically to understand their clinical significance. The most promising strategy to establish herd immunity against SARS-CoV-2 and its emerging variants is the development of efficient vaccines. In the present compilation, the zoonotic spread of SARS-CoV-2 and developments in the field of viral vector-based COVID-19 vaccines are discussed, with special emphasis on the status of current developments in the field of replicating viral vector-based vaccines for COVID-19.

## 2. Zoonotic Spread of SARS-CoV-2

**Figure 1:**
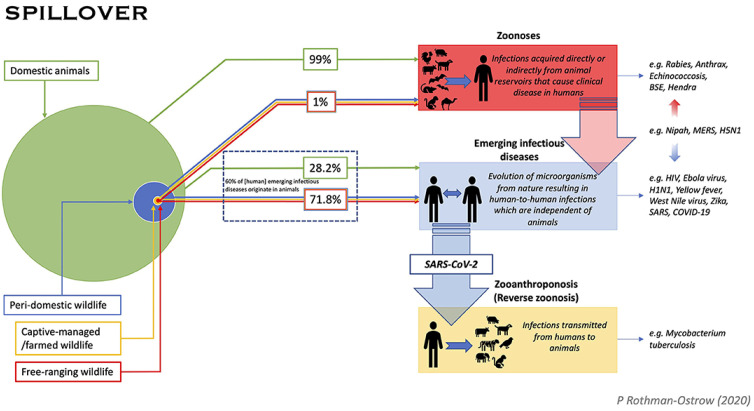
Pathway diagram for pathogen spill-over to humans from animals describes three distinct processes. (1) Zoonoses: pathogens that are transmitted from an animal reservoir directly or indirectly (e.g., foodborne, vector-borne, etc.) to humans, causing disease; (2) Emerging infectious diseases: pathogens that cause an emergent infectious disease in humans and persist in human populations irrespective of an animal reservoir. Genetic origins may show links to non-human animals, but these diseases undergo a more complex process of evolution not necessarily dependent on a specific animal reservoir, and usually evolve to be independent of animals; (3) Zooanthroponosis: reverse zoonosis whereby humans transmit infection to animals. (Reproduced from [33] under the terms of the Creative Commons Attribution License (CC BY)).

The origin of SARS-CoV-2 is among the most sought-after answers related to the COVID-19 pandemic [23]. The most controversial theories, however, are related to the lab-based origins of the virus. From the intentional engineering of biological weapons to the accidental spillover of the virus while working in the laboratory, a plethora of speculations revolves around the assumption that the virus is lab-made [24]. Although a very questionable notion to believe in, the theory of the manmade or accidental origin of the deadly virus also exists [25]. However, the validity of the proposed theory is substantially based on the fact that the Wuhan Institute of Virology in the city of Wuhan in China was coincidently working on a group of beta-coronaviruses isolated from bats, at the time of virus emergence [26]. One of the most commonly talked-about stories of the lab-based origin of the novel coronavirus is that the virus was intentionally (or accidentally) engineered for the gain of function studies on the pre-existing SARS-CoV that, unfortunately, resulted in virus spillover in humans; although the worked upon beta coronaviruses isolated from the bats in the concerned lab were only a very distant relative of the concurrently emerging SARS-CoV-2 [27,28,29]. Moreover, inferences from different studies indicate that it is very improbable that the present virus is engineered [30]. The features of the receptor-binding domain of SARS-CoV-2 differ from the ideal (optimal) receptor-binding domain. Further, though studies suggest that SARS-CoV-2 binds to the human ACE2 receptor with high affinity, the interaction does not appear to be ideal as per the computational analysis [31,32]. Figure 1 represents the possible pathways that led to the viral spillover from different potential sources.

**Figure 2:**
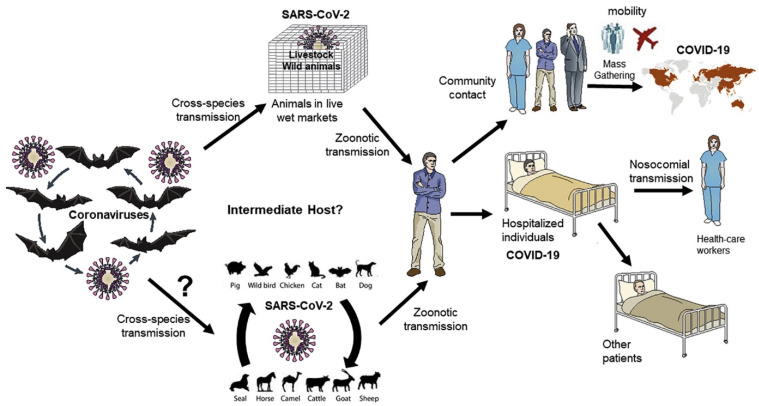
The emergence of SARS-CoV-2 and the outbreak of COVID-19. (Reproduced from [59] under the terms of the Creative Commons Attribution License (CC BY)).

On the other hand, the zoonotic origin of SARS-CoV-2 is the most well documented. Sequencing of the initially isolated novel SARS-CoV-2 in early 2020, showed that the nearest coronaviruses bearing the sequence similarity are found in two different strains of bats (bat-SL-CoVZXC21 and bat-SL-CoVZC45) from which the similar (although not the same) coronaviruses were isolated in the year 2015 and 2017, respectively [34,35]. The isolated viruses were almost 88% identical to the SARS-CoV-2. Surprisingly, the novel SARS-CoV-2 showed lesser sequence similarities with its predecessors: SARS-CoV (79%) and MERS-CoV (50%) [36,37]. Another convincing factor that favors the zoonotic origin of the virus is the history of coronavirus infection in around 500 species of chiropterans (consisting of more than 1200 species of bats), that ultimately serve as the reservoir for the evolution of different coronaviruses [38,39,40]. Undoubtedly, co-infection of CoV family members in the host must have paved novel routes for the recombination of the viral genome to produce newer strains [41]. Although reports are scarce to suggest direct transmission routes of coronaviruses from bat to humans, available investigations are suggesting the infectious propensity of chiropteran CoV in humans, at least in different cultured human cells [38,42]. In similar studies, the virus was found to be 96% genetically identical to a coronavirus isolated from bats [43,44]. Moreover, genetic recombination is a commonplace phenomenon observed in chiropteran isolated CoVs [45]. Considering this, it should occasion no surprise in surmising that the novel second version of SARS-CoV carries a botched-up genome, that is comprised of pieces of genomic sequences from pre-existing CoVs (Figure 2).

Another proposed possibility of the virus origin links with pangolins [46]. Reports of initial cases of COVID-19 are linked to the Huanan market of Wuhan, that serves as the source of almost all sorts of “eatable” animals [47]. It is possible that the first source of the lethal virus was present in the market, including the most notorious horseshoe bat (*Rhinolophus affinis*) [43,48]. However, there is one discrepancy to consider before accepting the bats as the most potent source of virus transmission to humans. RaTG13, isolated from the horseshoe bat shows deviation from the receptor-binding domain (RBD) of SARS-CoV-2 [31,48]. On the other hand, CoVs isolated from pangolins show considerable similarity to the RBD of SARS-CoV-2 [49]. In that view, if originated from a bat, it is very likely that the virus transmitted to another carrier (maybe a pangolin) where it underwent substantial genomic recombination before its ultimate transmission into human hosts [50].

Despite numerous reports inclined towards the zoonotic origin of the virus, one cannot simply sideline the fact that no animal originated CoV is thus far identified which is considered similar to the initial edition of SARS-CoV-2; however, under-sampling of the diverse variety of coronaviruses remains to be a concern [51]. Following this, Anderson and coworkers in their opinion article, “The proximal origin of SARS-CoV-2”, proposed another possibility wherein the virus was first transmitted to humans (from a zoonotic source), where it had many opportunities to mutate and evolve in a large set of the population, to let the process of natural selection proceed smoothly. In such a case, the virus would have developed the additional features (including the receptor-binding domain and polybasic cleavage site) “silently” during the initial human-to-human transmission [52]. Once achieved, the virus then possibly spread in large clusters to produce symptoms that ultimately came under human detection [48] (Figure 2). SARS-CoV-2 has mutated over time and several genetic variants have been reported to date including Omicron [53,54,55]. Recently, Wei C and coworkers, from the Chinese Academy of Sciences, reported that Omicron’s progenitor jumped from humans to mice, a reverse zoonotic event (in mid-2020), and accumulated mutations in a mouse host before jumping back to humans [50]. The Omicron variant has significantly more mutations compared to previous SARS-CoV-2 variants [56,57,58]. The study demonstrated that the molecular spectrum of mutations in Omicron has displayed prominent dissimilarities with the molecular spectra of variants that evolved in humans, and this variant has closely resembled the mutations associated with virus evolution in mouse cells [50].

Taken together, while different theories provide logical explanations for the origin of the deadly virus, more scientific evidence is required to understand the possible links to decipher the convoluted route of virus transmission from natural sources to humans.

## 3. Viral Vector-Based Vaccines—Historical Perspective

Obtaining wide public health advantages from immunization necessitates efficient policies that support vaccine development, assure vaccine finance, and increase vaccine access. Immunization trust is based on trust in the vaccines’ safety and effectiveness, confidence in vaccine producers and the physicians who deliver vaccinations, and trust in authorities who evaluate scientific data and issue immunization guidelines [60]. According to Shimada and colleagues [61], “Viral vectors are promising tools for vaccines. Viral vector-based vaccines can enhance immunogenicity without an adjuvant and induce a robust cytotoxic T lymphocyte (CTL) response to eliminate virus-infected cells. During the last several decades, many types of viruses have been developed as vaccine vectors. Each has unique features and parental virus-related risks. In addition, genetically altered vectors have been developed to improve efficacy and safety, reduce administration dose, and enable large-scale manufacturing.” Viral vector-based vaccines are not new but they were introduced for use in 1972 [62]. The advantages of viral vectors are as follows: “(a) high-efficiency gene transduction; (b) highly specific delivery of genes to target cells; and (c) induction of robust immune responses and increased cellular immunity.” The safety and effectiveness of viral vector-based vaccines must be evaluated, covering immunogenicity, genetic stability, the capacity to escape pre-existing defense, replication deficit or suppression, and genotoxicity. In addition, because infectious diseases are a problem in developing nations, cost-effectiveness must be assessed [62]. As a result, large-scale viral vector production must be considered. Generally, viral vectors are created by propagating the appropriate cell lines. In 1990, the first clinical trial of a therapeutic retroviral vector was conducted. Subsequent clinical trials have raised major concerns about genotoxicity, owing mostly to the possibility of viral genome integration. As the adeno-associated virus (AAV) vector may express episomal genes without integrating into the host genome, it has been cleared for clinical use by the EMA [63].

Currently, several viral vectors are being researched. As previously stated, each vector offers distinct advantages. Taking advantage of their benefits will improve their potential and speed up the clinical deployment of viral vector-based vaccinations. These vaccines can elicit a strong immune response in tissues and cells, while also achieving targeted delivery. Early-stage trials demonstrate that they are well tolerated in humans. Efforts to establish and optimize vaccine regimens will continue.

Vaccines targeting COVID-19 have been developed using a diverse range of viral vectors. In both rats and primates, adenovirus-based vectors elicited significant immune reactions and tolerance against SARS-CoV-2 exposures. Furthermore, protection was established in rodents after immunization with the lentivirus, measles viruses, Newcastle disease virus, and vesicular stomatitis virus vectors. The traditional route of administration is intramuscular, although intranasal applications have shown potential for adenovirus, LV, and influenza viral vectors. Table 1 summarizes the list of authorized viral vector-based vaccines of COVID-19. All are non-replicating viral vector-based vaccines based on adenovirus, while there are many replicating viral vector-based vaccines under clinical development.

## 4. Viral Vector-Based Vaccines for COVID-19

In the present situation, vaccines based on viral vectors have emerged as the leading candidates for developing an effective, safe, and mass-producible vaccine to combat the ongoing COVID-19 pandemic [73]. This vaccine platform employs replicating and non-replicating viral vectors. As evidenced by the triumph of the smallpox vaccine, Ervebo, and other potential vaccines against numerous infectious diseases, viral vectors present an appealing platform for COVID-19 vaccine development [74]. As per the WHO draft landscape of COVID-19 vaccine candidates, as of 25 January 2022, 23 viral vector-based vaccine candidates are currently in clinical trials (19 non-replicating and 4 replicating) [15]. COVID-19 vaccine development used diverse vectors, including MVA, Ad, Parainfluenza viruses, Sendai viruses, Rabies viruses, Newcastle viruses, and Influenza viruses [74]. For the administration of the majority of vaccine candidates, the intramuscular route is the most preferable one. Table 2 encompasses the pros and cons of replicating and non-replicating viral vectors for vaccine delivery.

The replicating vector vaccines infect host cells, resulting in vaccine antigens and new viruses that can evoke immunogenicity and transmit the infection by infecting healthy cells [75]. The recently approved Ebola vaccine is a viral vector vaccine that replicates within cells. These vaccines are generally harmless and elicit a robust immune response. However, the effectiveness of the vaccines could be reduced by pre-existing immunity to the vector [76]. Non-replicating vector vaccines, on the other hand, after infecting the host cells can produce vaccine antigens but do not yield new virus particles. To elicit persistent immunity, booster shots may be essential. Johnson & Johnson, a pharmaceutical company based in the United States, is working on this strategy [75,76].

As of 25 January 2022, 10 vaccines against COVID-19 have been approved by WHO, three of which are non-replicating viral vectors, viz., Ad26.COV2.S, AZD1222, and Covishield. These vaccines have been considered under WHO emergency listing, after assuring safety and efficacy through several clinical trials [16]. However, as stated by WHO, all these vaccines possess some common side effects such as itching, headaches, joint pain, a feverish sensation, muscular pain, vomiting, swelling at the injection site, and also flu-like symptoms such as runny nose, coughs, high temperature, sore throat, and so on.

Janssen (Johnson & Johnson) created Ad26.COV2.S, a non-replicating human adenovirus type 26 (Ad26) vector that expresses a pre-fusion stabilized SARS-CoV-2 spike protein from the Wuhan 2019 strain that is similar to the WA1/2020 spike protein. During preclinical and clinical studies, Ad26.COV2.S established defending efficacy against SARS-CoV-2 infection in hamsters and nonhuman primates, as well as safety and immunogenicity in humans, respectively [77,78]. Ad26.COV2.S has approval from authorities such as the Caribbean Regulatory System Emergency Use Recommendation, Africa Regulatory Taskforce Endorsement, and WHO Emergency Use listing and is currently undergoing immunization in 106 countries globally. The “thrombosis with thrombocytopenia syndrome” is a rare serious adverse event of Ad26.COV2.S [16]. However, a single dose of this vaccine is still considered efficacious and a reasonable choice for countries, particularly when faced with supply shortages and difficult-to-reach populations, the WHO recommends a two-dose regimen whenever feasible. According to WHO, the second dose should be given 2–6 months after the first. The COVID-19 epidemiological scenario, vaccine equipment, and needs for particular subpopulations all influence the inter-dose interval. Nevertheless, in clinical trials, this vaccine was found to be effective against the B1.351 and P.2 variants of the SARS-CoV-2 virus [79].

Another approved vaccine is Vaxzevria, also referred to as AZD1222, ChAdOx1 nCoV-19, which is a non-replicating vaccine based on a viral vector designed by the University of Oxford, in association with AstraZeneca. Following the rise of SARS-CoV-2, ChAdOx1 MERS, one of the promising vaccine candidates for MERS-CoV, was repurposed, and AZD1222 was designed to encode a full-length codon-optimized S protein of SARS-CoV-2. ChAdOx1 is a non-replicating simian adenoviral vector resulting from isolate Y25 [80]. According to Dicks et al., in the human population, the seroprevalence of antibodies to Y25 is 9% in the Gambia and 0% in the United Kingdom [81]. This vaccine has been authorized in 137 countries by various authorities [16]. A unique new type of adverse event, recognized as Thrombosis with Thrombocytopenia Syndrome (TTS), encompassing unusual and extreme blood clotting events related to low platelet counts, has been observed following vaccination with this vaccine. The vaccine had a 76% efficacy against symptomatic SARS-CoV-2 infection. However, this is only applicable to events occurring 15 days after the second dose, with a 29 day inter dose interval [82].

Covishield, an Indian version of AZD1222 developed by the Serum Institute of India, has been approved in 47 countries [83,84]. Abdominal pain, dizziness, decreased appetite, enlarged lymph nodes, excessive sweating, itchy skin, or rashes are some of the uncommon side effects of Covishield [85].

Sputnik V (Gam-COVID-Vac), a non-replicating adeno viral vector-based vaccine developed by the Gamaleya Research Institute of Epidemiology and Microbiology, was named after the first artificial satellite. The vaccine was developed as two formulations (frozen (Gam-COVID-Vac) (storage temperature −18 °C (0 °F)) and lyophilized (Gam-COVID-Vac-Lyo) (storage temperature 2–8 °C (36–46 °F)) [83]. Sputnik V has been authorized in 71 countries, but the World Health Organization and the European Medicines Agency have yet to approve it. As per WHO officials, the Sputnik V manufacturing process did not meet the required standards, and the EMA review has been delayed because some data are still missing. Although, as shown in a recent Gamaleya Center laboratory study, the Sputnik V vaccine is also efficacious against Omicron, the super mutant and vaccine-evading variant of COVID-19 [86].

The Gamaleya Research Institute of Epidemiology and Microbiology developed another single dose Sputnik light vaccine based on the Ad26 vector, which has been approved under emergency authorization in 27 countries as of 26 January 2022 [87].

CanSino Biologics’ Convidecia is a single dose viral vector-based vaccine, comparable to AZD1222 and Sputnik V, that has been officially approved by China and is under emergency authorization in nine countries as of 26 January 2022 [87].

## 5. Replicating Viral Vector-Based COVID-19 Vaccines and Their Mechanism of Action

The viral vector-based vaccine is one of the advanced approaches for vaccine development among other approaches. It contains attenuated viruses that are unrelated to the disease, and they are modified as vectors such that when they deliver genetic material (DNA) to human cells, it creates viral protein against a particular pathogen. This is recognized as foreign matter by the body and it initiates the immune response against it, as in the case of the normal defense mechanism of the body [88,89]. The viral vector infects antigen-presentation dendritic cells and macrophages, increases co-stimulatory substances that act as adjuvants, and elicits cytokine and chemokine responses, efficiently exposing antigens to the immune cells and triggering robust immune systems [90,91].

### 5.1. Mechanism of Action

**Figure 3:**
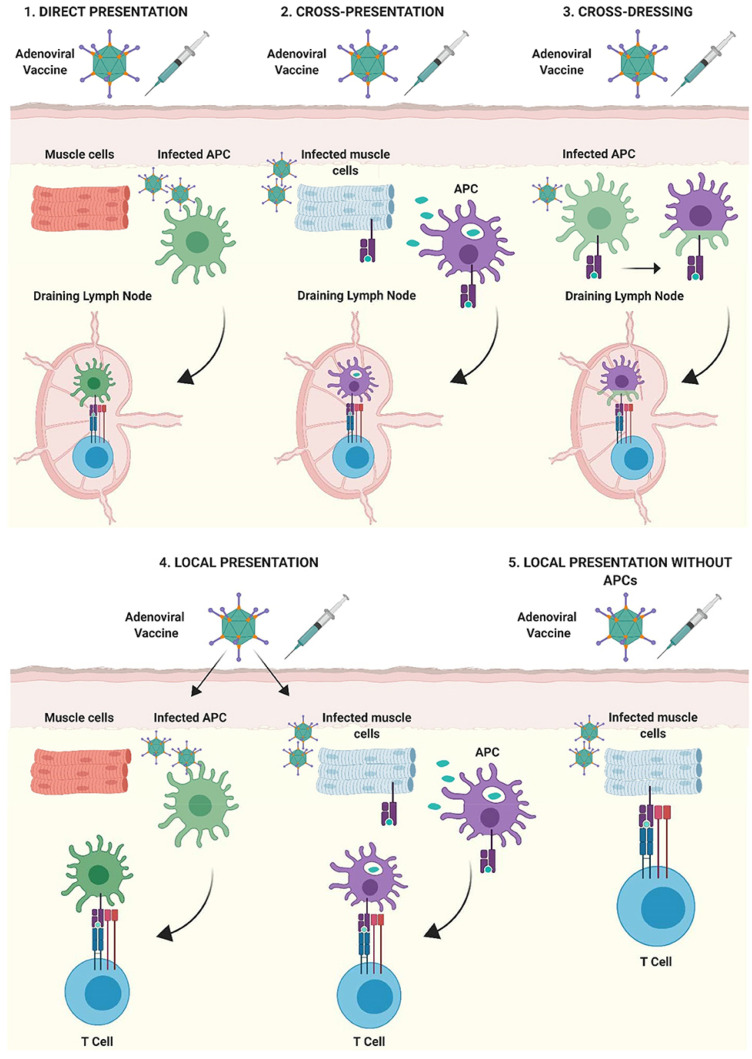
Various pathways of antigen presentation of viral vectored vaccine after intramuscular vaccination. (Adapted from Creative Commons Attribution License (CC BY from [94].).

There are two types of viral vector vaccines, non-replicating (replication-incompetent or replication-deficient) and replicating (self-replicating or replication-competent) viral vector-based vaccines. The replication-deficient viral vectors are modified such that they cannot create new viral entities, while replicating viral vector vaccines produce new viral entities once they infect the cells. The production of new viral entities occurs through the host cell mechanism, which then infects other host cells to produce additional viral antigens [88]. In replicating viral vector vaccines, only the E3 region of the genetic material of the viral vector is deleted, unlike non-replicating viral vaccines where E1 and E3 regions of the gene are deleted to create space for a foreign gene. However, this results in a limited replication capacity of 3–4 kb as compared to replication-defective viral vectors. Further, as the multiplication of viral vectors occurs in vivo, this results in enhanced antigen presentation that provokes strong responses. Thus, it exhibits the dose-sparing effect, which means even at a low dose, it indicates sufficient effect and maybe only a single dose can be sufficient to impart protection to the infection [89,92]. Moreover, the replicating viral vectors simulate the natural infection, so it induces cytokines and other stimulatory molecules that provide a potent adjuvant effect. Thus, these types of vectors can impart innate immunity, and humoral, cellular, and mucosal immune responses (Figure 3) [75].

For the COVID-19 vaccine, genetic material (DNA) for the S protein (spike protein) from the SARS-CoV-2 virus is modified and placed in a suitable unrelated viral vector, which when entering the cells, delivers the genetic material from the SARS-CoV-2 virus to the host cells. This instructs and provokes the body cells to make copies of the spike protein. When these copies of spike proteins are exposed on the cell surfaces, the body’s immune system responds to them by creating antibodies. Thus, if the person is infected with the COVID-19 virus, the antibodies will combat it. The viral vectors are made harmless through genetic modification and also the transferred genes do not become part of host DNA, hence they do not cause the individual to become infected with the viral vector virus or SARS-CoV-2 virus [93].

### 5.2. Replicating Viral Vaccines under Various Clinical Trials

There are many replicating and non-replicating SARS-CoV-2 candidate vaccines being studied in clinical trials [89]. The Janssen (Johnson & Johnson) COVID-19 vaccine and ChAdOx1 nCoV-19, developed by AstraZeneca and the University of Oxford, are examples of a non-replicating viral vector vaccines [95]. Different vaccine platforms have been utilized for the development of COVID-19 vaccines including viral vector vaccines. From them, Ad26COVS1 (Janssen/Johnson & Johnson), Vaxzevria (Oxford/AstraZeneca), and Covishield (Oxford/AstraZeneca formulation) have been approved by WHO and have displayed safety and efficacy against severe critical diseased conditions [71,77,96]. However, these vaccines may be associated with a rare risk of thrombotic events associated with thrombocytopenia [97].

There have been examples of preclinical trials of replicating viral vector-based vaccines suggesting their effectiveness in non-coronavirus candidates. The University Health Network, Canada has performed preclinical trials of replicating viral vector-based vaccines for recombinant measles virus with viral spike protein [98]. The Pasteur Institute has also worked on a MV-SARS recombinant measles virus vaccine, expressing SARS-CoV antigen against SARS in their preclinical studies, which have shown prominent efficacy in a non-coronavirus candidate, such as West Nile virus, CHIK virus, Ebola virus, Lassa virus, Zika virus, and is now under phase III clinical trial [99].

Besides the non-viral vector-based vaccine, replicating viral vector-based vaccines for COVID-19 have also been developed by various countries that are under various phases of clinical trials and have indicated promising results (Table 3).

As is evident from Table 3, three vaccines are in phase II, while two vaccines have reached phase III trials. The detailed study protocol of these vaccines and their outcome are discussed in the following section. Currently, there are eight replicating viral vector vaccines under clinical development including Brilife, DelNS1-2019-nCoV-RBD-OPT, AV-COVID-19, AdCLD-CoV19, COH04S1, Covid-19/aAPC, MV-014-212, and DelNS1-nCoV-RBD LAIV [100]. These vaccines are under different phases of development to assess their safety and efficacy. AdCLD-CoV19, a vaccine candidate of Cellid, displayed no difference between middle and high doses in the phase IIa trial. The high dose group had a 1.4 times higher neutralizing antibody titer than that of the middle dose group. All solicited adverse events were found in the category of grade 2 or lower for seven days after vaccination. However, they did not disclose unsolicited adverse events. Furthermore, Cellid announced plans to newly develop AdCLD-CoV19-1, creating a revised version of the virus vector vaccine [101].

The Israel Institute for Biological Research (IIBR) produced the replicating recombinant VSVΔG-spike vaccine named ‘Brilife’ (IIBR-100), which has been studied in phase I/II of a clinical trial (NCT04608305). The mechanism of action involves the replacement of the glycoprotein (G) gene of VSV (vesicular stomatitis virus) by the spike protein of the SARS-CoV-2 virus. The aim was to evaluate the immunogenicity, potential efficacy, and safety of an rVSV-SARS-CoV-2-S vaccine. The study incorporated two phases, wherein in the phase I (dose-escalation phase), 1040 subjects were enrolled in the age group of 18–55 years and they were randomly allocated a single dose of IIBR-100 at a low, mid, or high dose, or saline, or two administrations of IIBR-100 at a low dose, or saline with a gap of 28 days. Phase II trials were carried out with larger groups based on data reviewed in phase I, where they were also randomly allocated and given a similar dose treatment. Based on immunogenicity responses, the booster dose was implemented [102].

NeuroRx, Inc. also conducted a phase IIb/III (NCT04990466) study of VSV-ΔG SARS-CoV-2 vaccine in collaboration with Cromos, Brilife Georgia, and the Israel Institute for Biological Research, with the incorporation of 550 subjects in the year 2021. This clinical trial was performed to support late-stage clinical studies and the subsequent mass immunization of the Georgian population. In this randomized, multi-center, and observer-blind study, the subjects received two intramuscular injections of the vaccine (prime-boost) with a gap of 28 days. It consisted of 1 mL replicating viral rVSV-SARS-CoV-2-S vaccine or an active comparator and periodical follow up was undertaken up to 12 months. Pre-clinical data suggested IIBR-100 to be an efficacious and protective vaccine against SARS-CoV-2 infection and it has shown no signs of safety concerns [103].

Wantai Biopharm, China carried out a phase I clinical trial (ChiCTR2000037782) of an influenza virus vector COVID-19 vaccine (DelNS1-2019-nCoV-RBD-OPT1) to be administered as an intranasal spray on 60 healthy subjects. The purpose of the study was the evaluation of its safety as well as the determination of the influence of pre-existing antibodies against influenza A (H1N1) virus (A/California/4/2009, CA4) on the immunogenicity of the influenza virus vector COVID-19 vaccine. Moreover, the virus excretion of the vaccine strain post-vaccination was also studied. A parallel phase II clinical trial of DelNS1-2019-nCoV-RBD-OPT1 vaccine (intranasal spray) was carried out in China (ChiCTR2000039715) on 720 healthy subjects. The objective of the study was to evaluate the immunogenicity and safety of the influenza virus vector COVID-19 vaccine for intranasal spray (DelNS1-2019-nCoV-RBD-OPT1), according to different immunization procedures. In addition, the effects of pre-existing H1N1 influenza virus (A/California/4/2009, CA4) antibodies on the immunogenicity of the vaccine, according to different immunization procedures, were also investigated. The study has also undergone the phase III trial (ChiCTR2100051391) in the Philippines involving 40,000 subjects [104,105,106].

Cellid Co., Ltd., Seoul, Republic of Korea initiated a phase I/IIa clinical trial (NCT04666012) in the year 2020 on 150 healthy volunteers to assess the immunogenicity and safety of the AdCLD-CoV19 vaccine. In the study, part A of phase I was conducted as single-center, dose-escalation, and open-label study, while part B of the phase IIa clinical trial was carried out as an open-label, multi-center study. The safety in all the dose groups was assessed in part A and two suitable doses were set for part B in which the immune response and safety were evaluated against SARS-CoV-2. Based on the interpretations, a suitable dose for the next phase of the clinical trial was determined [107].

Aivita Biomedical, Inc. (Irvine, CA, USA), in collaboration with PT AIVITA Biomedika Jakarta, Indonesia, Kariadi Hospital, and Central Army Hospital RSPAD Gatot Soebroto, initiated a phase II study (NCT05007496) of a preventive dendritic cell vaccine, AV-COVID-19 (anti-SARS-CoV-2 COVID-19 vaccine). This randomized, double-blind study was performed in the year 2021 on 145 subjects who were not actively infected with COVID-19. In the study, AV-COVID-19, which was made on-site using PT AIVITA Biomedika Indonesia’s vaccine-enabling kit, was tested against COVID-19 infection. The vaccine consists of autologous dendritic cells and lymphocytes (DCL) which were incubated with a quantity of SARS-CoV-2 S protein (spike protein). This is a subject-specific personal vaccine that proved to be safe in the phase I trial conducted in Indonesia. The safety and efficacy of the vaccine was also assessed in a phase II clinical trial wherein the improved T cell response specific to the S protein was evaluated by comparison of the results before and up to 4 weeks post-vaccination. Observations for any serious adverse events and situations demanding immediate medical intervention were also made for 2 months post-vaccination [108].

Aivita Biomedical, Inc, has also proposed an adaptive phase I/II clinical trial (NCT04386252) in the United States of America to be initiated in 2023. It includes a vaccine comprising autologous dendritic cells previously incubated with S protein from SARS-CoV-2, with or without GM-CSF (granulocyte-macrophage colony-stimulating factor), in 175 subjects. The subjects selected were negative for COVID-19 infection as well as for anti-SARS-CoV-2 antibodies to prevent COVID-19 infection in adults. The subjects were tested through nasal swabs to exclude subjects actively infected with COVID-19. The quality testing and safety assessment will be carried out using a small quantity of the batch [109].

“Indonesia-MoH in collaboration with Aivita Biomedical, Inc.; PT AIVITA Biomedika Indonesia; the National Institute of Health Research and Development, Ministry of Health Republic of Indonesia; RSUP Dr. Kariadi Semarang, Indonesia; and the Faculty of Medicine, University of Diponegoro, Indonesia commenced an adaptive phase I clinical trial of a preventive vaccine. It consists of autologous dendritic cells, which were incubated previously with S protein from SARS-CoV-2 without GM-CSF, in 27 subjects who are negative for COVID-19 infection and anti-SARS-CoV-2 antibodies (NCT04685603, NCT04690387) [110,111].”

The Health Institutes of Turkey, in collaboration with TC Erciyes University, also carried out a phase I trial (NCT04691947) (double-blind, double dose, parallel, randomized vaccination) of ERUCOV-VAC (hCoV-19/Turkey/ERAGEM-001/2020) (whole-virion inactivated SARS-CoV-2 vaccine) vaccine on 44 subjects, with the aim of investigating the safety and immunogenicity of two different strengths (3 µg and 6 µg) of an inactivated COVID-19 vaccine compared to a placebo to demonstrate the safety and efficacy in prophylaxis of COVID-19. It is also being studied in phase II clinical trials (NCT04824391) on 250 participants [112,113]. The vaccine will be named TURKOVAC in the phase III clinical trial [114].

The City of Hope Medical Center, United States of America, commenced the phase I study (NCT04639466) of a synthetically modified vaccinia Ankara- (MVA) based SARS-CoV-2 vaccine, COH04S1, on 189 volunteers to prevent COVID-19 infection. The purpose of this study is to determine the optimal dose of the COH04S1 vaccine and to assess its safety. COH04S1 is a synthetic version of MVA, a new version of modified vaccinia Ankara. The mechanism by which COH04S1 works is through the induction of immunity to the SARS-CoV-2 virus. COH04S1 was produced by placing small pieces of SARS-CoV-2 DNA into synthetic MVA, which may be able to induce immunity to SARS-CoV-2 by creating antibodies against it. This inhibits the virus from entering healthy cells and the immune system also develops T cells which can recognize and destroy infected cells. The administration of COH04S1 after cellular therapy may show better results in reducing the chances of COVID-19 infection or of the infection evolving into a severe form of COVID-19 disease in blood cancer patients, as compared to Emergency Use Authorization (EUA) SARS-CoV-2 vaccine. The dose-escalation study was performed to evaluate the safety and to determine the effective biological dose of vaccine COH04S1, through administration of one or two injections, or as a booster to healthy adult volunteers. A multi-center, observer-blinded, EUA vaccine-controlled, randomized phase II trial (NCT04977024) of COH04S1 vaccine was also initiated to determine the immune response to COH04S1 vaccine as compared to the EUA SARS-CoV-2 vaccine. This was performed in patients with blood cancer who have received cellular therapy (HCT or CAR-T) or stem cell transplants. Further, the safety aspects of 240 participants were also assessed [115,116].

## 6. Drug Delivery Route and Delivery Systems

The eminence of the generated response through viral vector vaccines also depends upon the route of administration and the delivery system [117]. The different routes through which viral vectors can be administered are intramuscular, intradermal, intranasal, and oral routes, as evident from the clinical trials for various viruses [117]. The various delivery systems are also being developed to impart the maximum efficacy and safety of the developed vaccines and are discussed below:

Intravenous route: Despite being the most common route of administration for drug delivery, the intravenous route is not the favorable route for immunization specifically in a pandemic. The replicating viral vector vaccines, AdCLD-CoV19, developed by Cellid Co., Ltd., and COH04S1, by the City of Hope Medical Center, United States of America, are intended for the parenteral route in the form of injection [118].

Intramuscular route: Most of the viral vector vaccines for COVID-19 are formulated to be administered through the intramuscular route [119]. The recently developed replicating viral vector-based vaccines for COVID-19, such as Brilife by the Israel Institute for Biological Research [103], and ERUCOV-VAC by the Health Institutes of Turkey [112], are based on the intramuscular route.

Subcutaneous route: With the acceptance of the intramuscular route, researchers have also developed the replicating viral vector vaccine to be administered by subcutaneous administration, such as AV-COVID-19, by the Aivita Biomedical, Inc., Indonesia [111].

Intranasal route: For mucosal response, the nasal route is preferable to the parenteral route, keeping in view its reliability and ease of application [9,117,120]. Further, to prevent SARS-CoV-2 virus replication in nasal epithelia, the local mucosal immune response is significant and so, many pharmaceutical companies and researchers have developed mucosal formulations for the COVID-19 vaccine, although the mucosal route is not the common route of administration for immunization and the nasal route is preferred more for imparting direction action in COVID-19. The vaccine BBV154, developed by the Washington University School of Medicine in St. Louis, USA, and Bharat Biotech, India, is an example of an intranasal non-replicating viral vector-based vaccine that has completed phase I clinical trial (NCT04751682). The most favorable among all vaccines is the replicating viral vector-based vaccine, DelNS1-2019-nCoV-RBD-OPT1, by Wantai Bio-Pharm, China, which is to be administered as an intranasal spray which may be valuable for easy administration and patient compliant vaccination in outbreak situations [104,121].

Oral route: In addition to the intranasal mucosal routes, oral vaccine VXA-CoV2-1.1-S in the form of a tablet, by Vaxart, USA, is under phase II clinical trials (NCT05067933) and another capsule-based oral formulation named OraPro-COVID-19, has been developed and studied by iosBio Pharma in the UK in collaboration with BioCell Corporation, Auckland, New Zealand, aiming at rapid deployment of vaccination [122,123].

Thus, as for the conventional vaccine delivery system, the parenteral route is the most preferred route of administration, although its invasive nature and requirement for strict cold chain storage necessitate the non-invasive routes of immunization as well as the novel drug delivery systems [119]. Therefore, liposomes, nanoemulsions, and patches based on the micro needle are being investigated in preclinical studies, which may be proven as viable alternatives to invasive techniques [119]. The lipidic nanoparticles have proven exceptional applications in delivering the mRNA vaccine for COVID-19 infection, as evident from Moderna and BioNTech/Pfizer [124]. Moreover, a novel silica nanoparticle, Nuvec^®^, has the potential to be administered as a viral vector or as a delivery system for the transfer of genetic material into host cells [125].

## 7. Conclusions and Future Prospects

The SARS-CoV-2 outbreak has demonstrated that viral vector-based vaccines are promising vaccine options. Clinical trials utilizing viral vector-based vaccinations have shown that they are safe in humans, with the vast majority of people experiencing no serious adverse reactions. These same experiments have demonstrated the ability of viral vector-based vaccines to elicit powerful protective humoral immunity, even after a single dose in some situations. These findings show that viral vectors are among the vaccination platforms with the most potential in the fight against this pandemic. The multiplication of replicating viral vectors occurs in vivo; this results in enhanced antigen presentation that provokes strong responses. Thus, they exhibit the dose-sparing effect, meaning that even at a low dose, a sufficient effect is indicated and maybe only a single dose can be sufficient to impart protection to the infection. Moreover, the replicating viral vectors simulate the natural infection, so, they induce cytokines and other stimulatory molecules that provide a potent adjuvant effect. Thus, these types of vectors can impart innate immunity, and humoral, cellular, and mucosal immune responses. The remarkable improvements in vaccination technology and the greater cultural understanding of the value of vaccines in the healthcare system have provided a little positive side to this catastrophe. Even though the authors believe that the availability of increasingly effective and safe vaccinations allows for rapid deployment, this will inevitably stall in the absence of an ongoing pandemic.

## Figures and Tables

**Table 1:** List of authorized viral vector-based vaccines of COVID-19.

Vaccine Name	Viral-Vector Used	Manufacturer	Route/Dose	Efficacy	References
Vaxzevria Or Covishield	Chimpanzee adenovirus ChAdOx1 (Non-replicating)	Oxford University in collaboration with AstraZeneca.	Intramuscular injection (IM)/0.5 mL two doses of vaccine. Currently, the requirement for a booster dose.	76.0% effective at preventing symptomatic COVID-19 commencing 22 days from the first dose and 81.3% effective after the second dose. 81% and 61% effective against the B.1.1.7 and B.1.617.2 variants, respectively, after the second dose. Also effective for B.1.351.	[64,65]
JNJ-78436735	Human adenovirus (Ad26) (Non-replicating)	Janssen (Johnson & Johnson)	IM/0.5 mL single dose. Currently, the requirement for a booster dose.	66% effective in preventing symptomatic COVID-19 in a one-dose regimen 28 days after completion, with an 85% efficacy in preventing severe COVID-19 and a 100% efficacy in preventing hospitalization or death caused by the disease. Also effective for B.1.1.7 variant, B.1.351variant and P.2 variant.	[66]
Sputnik V (Gam-COVID-Vac)	Adeno (Ad26) viral vector (Non-replicating)	Gamaleya Research Institute of Epidemiology and Microbiology	IM/0.5 mL two doses. Currently, the requirement for a booster dose.	After the second dose efficacy is 91.6% for all age groups; about 90% effective against the B.1.617.2 variant. However, there was a noticeable decrease in neutralizing antibodies against B.1.351, P.1, and B.1.1.28 variants.	[67,68]
Sputnik light	Adeno (Ad26) viral vector (non-replicating)	Gamaleya Research Institute of Epidemiology and Microbiology	IM/0.5 mL single dose. Currently, the requirement for a booster dose.	The single-injection vaccine is 79% effective; 88% effective in preventing hospitalization, and 85% in preventing death (as per an Argentinian study with 60–79-year-old subjects). According to the Gamaleya Center, it is effective against all new variants.	[69,70]
CONVIDECIA (Ad5-nCoV)	Adeno (Ad5) viral vector (Non-replicating)	CanSino Biologics and the Beijing Institute of Biotechnology of the Academy of Military Medical Sciences.	IM/0.5ml single dose. Currently, the requirement for a booster dose.	65.7% efficacy in preventing moderate symptoms of COVID-19, and 91% efficacy in preventing severe disease. There is currently no clear information on variant efficacy.	[71,72]

**Table 2:** Pros and cons of replicating and non-replicating viral vectors.

Viral Vector	Pros	Cons
**Non-Replicating Viral Vector**		
Adenovirus	Safe. Stable genetically as well as physically. Infects both dividing and non-dividing cells, as well as dendritic cells. No integration. There are numerous serotypes and chimeric forms.	Prior Ad5 immunity. To elicit immunity, high doses are required.
Adeno-associated virus	Acid-resistance. Physical stability. Availability of alternative serotypes. Non-pathogenic.	Helper virus is required in production. Chances of integration. Prior immunity to common AAV2.
Alphavirus	No integration. Anti-vector immunity is not elicited. Dendritic cells are the target. Extremely immunogenic.	Safety concerns. Complicated to develop.
Herpesvirus	Infects a variety of cell types and primarily affects the mucosa. Long-lasting immunity. Th1 responses are induced.	Prior immunity. Reduced immunogenicity. Complicated to develop.
Poxviruses: NYVAC; MVA	Immunogenicity is remarkable.	Prior immunity.
Poxviruses: ALCAC; FPV	No prior immunity.	Immunogenicity is lower than that of mammalian poxviruses.
**Replicating Viral Vector**		
Adenovirus	Mucosal administration of a low dose. Immunity that lasts. Immune modulators are activated. As an oral vaccine, it is completely safe.	Insert size is small. Concerns regarding intranasal administration.
Measles virus	Long-lasting immunity. Infects dendritic cells and macrophages. No integration. Consistent genetically.	Prior immunity.
Poxviruses: Vaccinia	Extremely good immunogenicity and a track record of eradicating smallpox.	Concerns about safety in immunocompromised patients.
Vesicular stomatitis virus	No integration. The level of expression is high. Simple production. No prior immunity. Administration via mucosa.	Safe. Potentially neurovirulent. Immunogenicity is reduced in attenuated forms.

**Table 3:** Replicating viral vector-based COVID-19 vaccines under various stages of clinical development.

Vaccine	Developer	Country	Clinical Trial Registry No.	Clinical Trial Status	Viral Vector
Brilife (IIBR-100)	The Israel Institute for Biological Research (IIBR)	Israel	NCT04608305	Phase I/II	Vesicular stomatitis virus
	NeuroRx, Inc. in collaboration with Cromos, Brilife Georgia, Israel Institute for Biological Research	Georgia	NCT04990466	Phase IIb/III	Vesicular stomatitis virus
DelNS1-2019-nCoV-RBD-OPT1	Wantai Biopharm	China	ChiCTR2000037782	Phase I	H1N1 Influenza virus
		China	ChiCTR2000039715	Phase-II	H1N1 Influenza virus
		Philippines	ChiCTR2100051391	Phase III	H1N1 Influenza virus
AdCLD-CoV19	Cellid Co., Ltd.	Republic of Korea	NCT04666012	Phase I/IIa	Adenovirus
AV-COVID-19	Aivita Biomedical, Inc. in collaboration with PT AIVITA Biomedika Indonesia, Kariadi Hospital, Central Army Hospital RSPAD Gatot Soebroto	Indonesia	NCT05007496	Phase I/II	Autologous dendritic cells and lymphocytes (DCL)
	Aivita Biomedical, Inc.	United States of America	NCT04386252	Phase I/II	Autologous dendritic cells and lymphocytes (DCL)
	Indonesia-MoH in collaboration with Aivita Biomedical, Inc.	Indonesia	NCT04685603 NCT04690387	Phase I	Autologous dendritic cells and lymphocytes (DCL)
ERUCOV-VAC	The Health Institutes of Turkey in collaboration with TC Erciyes University	Turkey	NCT04691947	Phase I	Whole-virion inactivated
			NCT04824391	Phase II	Whole-virion inactivated
COH04S1	City of Hope Medical Center	United States of America	NCT04639466	Phase I	Synthetically modified vaccinia Ankara (MVA)
			NCT04977024	Phase II	MVA

## Data Availability

Not applicable.

## References

[1] Alamer F., Alamir A., AlJohani S., AlSumih N., Hiji F., Alhammadi M., Almuneef M. (2022). Childhood Vaccination Hesitancy in Saudi Arabia: A Time for Action. J. Infect. Public Health.

[2] Pollard A.J., Bijker E.M. (2020). A Guide to Vaccinology: From Basic Principles to New Developments. Nat. Rev. Immunol..

[3] Kaur N., Chaudhary V. (2021). Biotherapeutics and Its Applications in Microbiology. Environ. Conserv. J..

[4] Chavda V.P., Pandya R., Apostolopoulos V. (2021). DNA Vaccines for SARS-CoV-2: Towards Third Generation Vaccination Era. Expert Rev. Vaccines.

[5] Hilleman M.R. (2000). Vaccines in Historic Evolution and Perspective: A Narrative of Vaccine Discoveries. J. Hum. Virol..

[6] Plotkin S.A. (2003). Vaccines, Vaccination, and Vaccinology. J. Infect. Dis..

[7] Kallerup R.S., Foged C. (2015). Classification of Vaccines. Subunit Vaccine Delivery.

[8] Yadav D.K., Yadav N., Khurana S.M.P. (2020). Vaccines: Present Status and Applications. Animal Biotechnology.

[9] Chavda V.P., Vora L.K., Pandya A.K., Patravale V.B. (2021). Intranasal Vaccines for SARS-CoV-2: From Challenges to Potential in COVID-19 Management. Drug Discov. Today.

[10] Chavda V.P., Vora L.K., Vihol D.R. (2021). COVAX-19 Vaccine: Completely Blocks Virus Transmission to Non-Immune Individuals. Clin. Complement. Med. Pharmacol..

[11] Uddin M., Mustafa F., Rizvi T.A., Loney T., Al Suwaidi H., Al-Marzouqi A.H.H., Eldin A.K., Alsabeeha N., Adrian T.E., Stefanini C. (2020). SARS-CoV-2/COVID-19: Viral Genomics, Epidemiology, Vaccines, and Therapeutic Interventions. Viruses.

[12] Sharma O., Sultan A.A., Ding H., Triggle C.R. (2020). A Review of the Progress and Challenges of Developing a Vaccine for COVID-19. Front. Immunol..

[13] Emrani J., Ahmed M., Jeffers-Francis L., Teleha J.C., Mowa N., Newman R.H., Thomas M.D. (2021). SARS-COV-2, Infection, Transmission, Transcription, Translation, Proteins, and Treatment: A Review. Int. J. Biol. Macromol..

[14] Chavda V.P., Hossain M.K., Beladiya J., Apostolopoulos V. (2021). Nucleic Acid Vaccines for COVID-19: A Paradigm Shift in the Vaccine Development Arena. Biologics.

[15] WHO COVID-19 Vaccine Tracker and Landscape. https://www.who.int/publications/m/item/draft-landscape-of-covid-19-candidate-vaccines.

[16] WHO (2021). COVID19 Vaccine Tracker.

[17] Parums D. (2021). V Editorial: First Full Regulatory Approval of a COVID-19 Vaccine, the BNT162b2 Pfizer-BioNTech Vaccine, and the Real-World Implications for Public Health Policy. Med. Sci. Monit. Int. Med. J. Exp. Clin. Res..

[18] Tregoning J.S., Flight K.E., Higham S.L., Wang Z., Pierce B.F. (2021). Progress of the COVID-19 Vaccine Effort: Viruses, Vaccines and Variants versus Efficacy, Effectiveness and Escape. Nat. Rev. Immunol..

[19] Li C.-X., Noreen S., Zhang L., Saeed M., Wu P., Ijaz M., Dai D., Maqbool I., Madni A., Akram F. (2022). A Critical Analysis of SARS-CoV-2 (COVID-19) Complexities, Emerging Variants, and Therapeutic Interventions and Vaccination Strategies. Biomed. Pharmacother..

[20] Leung K., Wu J.T. (2021). Managing Waning Vaccine Protection against SARS-CoV-2 Variants. Lancet.

[21] Khan W.H., Hashmi Z., Goel A., Ahmad R., Gupta K., Khan N., Alam I., Ahmed F., Ansari M.A. (2021). COVID-19 Pandemic and Vaccines Update on Challenges and Resolutions. Front. Cell. Infect. Microbiol..

[22] Wilder-Smith A. (2021). What Is the Vaccine Effect on Reducing Transmission in the Context of the SARS-CoV-2 Delta Variant?. Lancet Infect. Dis..

[23] Hu B., Guo H., Zhou P., Shi Z.-L. (2021). Characteristics of SARS-CoV-2 and COVID-19. Nat. Rev. Microbiol..

[24] Nie J.-B. (2020). In the Shadow of Biological Warfare: Conspiracy Theories on the Origins of COVID-19 and Enhancing Global Governance of Biosafety as a Matter of Urgency. J. Bioeth. Inq..

[25] van der Linden S., Roozenbeek J., Compton J. (2020). Inoculating Against Fake News About COVID-19. Front. Psychol..

[26] Zhou P., Yang X.-L., Wang X.-G., Hu B., Zhang L., Zhang W., Si H.-R., Zhu Y., Li B., Huang C.-L. (2020). A Pneumonia Outbreak Associated with a New Coronavirus of Probable Bat Origin. Nature.

[27] Burki T. (2020). The Origin of SARS-CoV-2. Lancet. Infect. Dis..

[28] Rasmussen A.L. (2021). On the Origins of SARS-CoV-2. Nat. Med..

[29] Chavda V.P., Feehan J., Apostolopoulos V. (2021). A Veterinary Vaccine for SARS-CoV-2: The First COVID-19 Vaccine for Animals. Vaccines.

[30] Almazán F., Sola I., Zuñiga S., Marquez-Jurado S., Morales L., Becares M., Enjuanes L. (2014). Coronavirus Reverse Genetic Systems: Infectious Clones and Replicons. Virus Res..

[31] Wan Y., Shang J., Graham R., Baric R.S., Li F. (2020). Receptor Recognition by the Novel Coronavirus from Wuhan: An Analysis Based on Decade-Long Structural Studies of SARS Coronavirus. J. Virol..

[32] Sheahan T., Rockx B., Donaldson E., Sims A., Pickles R., Corti D., Baric R. (2008). Mechanisms of Zoonotic Severe Acute Respiratory Syndrome Coronavirus Host Range Expansion in Human Airway Epithelium. J. Virol..

[33] Haider N., Rothman-Ostrow P., Osman A.Y., Arruda L.B., Macfarlane-Berry L., Elton L., Thomason M.J., Yeboah-Manu D., Ansumana R., Kapata N. (2020). COVID-19—Zoonosis or Emerging Infectious Disease?. Front. Public Health.

[34] Lai C.C., Shih T.P., Ko W.C., Tang H.J., Hsueh P.R. (2020). Severe Acute Respiratory Syndrome Coronavirus 2 (SARS-CoV-2) and Coronavirus Disease-2019 (COVID-19): The Epidemic and the Challenges. Int. J. Antimicrob. Agents.

[35] Chavda V.P., Gajjar N., Shah N., Dave D.J. (2021). Darunavir ethanolate: Repurposing an anti-HIV drug in COVID-19 treatment. Eur. J. Med. Chem. Rep..

[36] Abdelrahman Z., Li M., Wang X. (2020). Comparative Review of SARS-CoV-2, SARS-CoV, MERS-CoV, and Influenza A Respiratory Viruses. Front. Immunol..

[37] Cui J., Li F., Shi Z.L. (2019). Origin and Evolution of Pathogenic Coronaviruses. Nat. Rev. Microbiol..

[38] Sallard E., Halloy J., Casane D., Decroly E., van Helden J. (2021). Tracing the Origins of SARS-COV-2 in Coronavirus Phylogenies: A Review. Environ. Chem. Lett..

[39] Lu R., Zhao X., Li J., Niu P., Yang B., Wu H., Wang W., Song H., Huang B., Zhu N. (2020). Genomic Characterisation and Epidemiology of 2019 Novel Coronavirus: Implications for Virus Origins and Receptor Binding. Lancet.

[40] Luk H.K.H., Li X., Fung J., Lau S.K.P., Woo P.C.Y. (2019). Molecular Epidemiology, Evolution and Phylogeny of SARS Coronavirus. Infect. Genet. Evol..

[41] Baddal B., Cakir N. (2020). Co-Infection of MERS-CoV and SARS-CoV-2 in the Same Host: A Silent Threat. J. Infect. Public Health.

[42] Menachery V.D., Yount B.L., Debbink K., Agnihothram S., Gralinski L.E., Plante J.A., Graham R.L., Scobey T., Ge X.Y., Donaldson E.F. (2015). A SARS-like Cluster of Circulating Bat Coronaviruses Shows Potential for Human Emergence. Nat. Med..

[43] Lau S.K.P., Luk H.K.H., Wong A.C.P., Li K.S.M., Zhu L., He Z., Fung J., Chan T.T.Y., Fung K.S.C., Woo P.C.Y. (2020). Possible Bat Origin of Severe Acute Respiratory Syndrome Coronavirus 2. Emerg Infect Dis..

[44] Paraskevis D., Kostaki E.G., Magiorkinis G., Panayiotakopoulos G., Sourvinos G., Tsiodras S. (2020). Full-Genome Evolutionary Analysis of the Novel Corona Virus (2019-NCoV) Rejects the Hypothesis of Emergence as a Result of a Recent Recombination Event. Infect. Genet. Evol..

[45] Hu B., Zeng L.P., Yang X.L., Ge X.Y., Zhang W., Li B., Xie J.Z., Shen X.R., Zhang Y.Z., Wang N. (2017). Discovery of a Rich Gene Pool of Bat SARS-Related Coronaviruses Provides New Insights into the Origin of SARS Coronavirus. PLoS Pathog..

[46] Frutos R., Serra-Cobo J., Chen T., Devaux C.A. (2020). COVID-19: Time to Exonerate the Pangolin from the Transmission of SARS-CoV-2 to Humans. Infect. Genet. Evol..

[47] Zhang T., Wu Q., Zhang Z. (2020). Probable Pangolin Origin of SARS-CoV-2 Associated with the COVID-19 Outbreak. Curr. Biol..

[48] Andersen K.G., Rambaut A., Lipkin W.I., Holmes E.C., Garry R.F. (2020). The Proximal Origin of SARS-CoV-2. Nat. Med..

[49] Zhang T., Wu Q., Zhang Z. (2020). Pangolin Homology Associated with 2019-NCoV. Curr. Biol..

[50] Wei C., Shan K.-J., Wang W., Zhang S., Huan Q., Qian W. (2021). Evidence for a Mouse Origin of the SARS-CoV-2 Omicron Variant. J. Genet. Genomics.

[51] Ye Z.-W., Yuan S., Yuen K.-S., Fung S.-Y., Chan C.-P., Jin D.-Y. (2020). Zoonotic Origins of Human Coronaviruses. Int. J. Biol. Sci..

[52] Seidah N.G., Pasquato A., Andréo U. (2021). How Do Enveloped Viruses Exploit the Secretory Proprotein Convertases to Regulate Infectivity and Spread?. Viruses.

[53] Basu D., Chavda V.P., Mehta A.A. (2022). Therapeutics for COVID-19 and Post COVID-19 Complications: An Update. Curr. Res. Pharmacol. Drug Discov..

[54] Chavda V.P., Apostolopoulos V. (2022). Omicron Variant (B.1.1.529) of SARS-CoV-2: Threat for the Elderly?. Maturitas.

[55] Chavda V.P., Kapadia C., Soni S., Prajapati R., Chauhan S.C., Yallapu M.M., Apostolopoulos V. (2022). A Global Picture: Therapeutic Perspectives for COVID-19. Immunotherapy.

[56] Chavda V.P., Apostolopoulos V. (2022). Global Impact of Delta plus Variant and Vaccination. Expert Rev. Vaccines.

[57] Chavda V.P., Patel A.B., Vihol D., Vaghasiya D.D., Ahmed K.M.S.B., Trivedi K.U., Dave D.J. (2022). Herbal Remedies, Nutraceuticals, and Dietary Supplements for COVID-19 Management: An Update. Clin. Complement. Med. Pharmacol..

[58] Chavda V.P., Apostolopoulos V. (2022). Is Booster Dose Strategy Sufficient for Omicron Variant of SARS-CoV-2?. Vaccines.

[59] El Zowalaty M.E., Järhult J.D. (2020). From SARS to COVID-19: A Previously Unknown SARS- Related Coronavirus (SARS-CoV-2) of Pandemic Potential Infecting Humans—Call for a One Health Approach. One Heal..

[60] Gerberding J.L., Haynes B.F. (2021). Vaccine Innovations—Past and Future. N. Engl. J. Med..

[61] Ura T., Okuda K., Shimada M. (2014). Developments in Viral Vector-Based Vaccines. Vaccines.

[62] Jackson D.A., Symons R.H., Berg P. (1972). Biochemical Method for Inserting New Genetic Information into DNA of Simian Virus 40: Circular SV40 DNA Molecules Containing Lambda Phage Genes and the Galactose Operon of Escherichia Coli. Proc. Natl. Acad. Sci. USA.

[63] Wang D., Tai P.W.L., Gao G. (2019). Adeno-Associated Virus Vector as a Platform for Gene Therapy Delivery. Nat. Rev. Drug Discov..

[64] Kandimalla R., Chakraborty P., Vallamkondu J., Chaudhary A., Samanta S., Reddy P.H., De Feo V., Dewanjee S. (2021). Counting on COVID-19 Vaccine: Insights into the Current Strategies, Progress and Future Challenges. Biomed..

[65] Chanukya G.V., Srikantam A. (2021). Comparative Quantitative Analysis of SARS-CoV-2 Spike Neutralizing Antibody Titers Following Two Anti COVID-19 Vaccines in India. medRxiv.

[66] Liu Q., Qin C., Liu M., Liu J. (2021). Effectiveness and Safety of SARS-CoV-2 Vaccine in Real-World Studies: A Systematic Review and Meta-Analysis. Infect. Dis. Poverty.

[67] Cazzola M., Rogliani P., Mazzeo F., Matera M.G. (2021). Controversy Surrounding the Sputnik V Vaccine. Respir. Med..

[68] Pouresmaieli M., Ekrami E., Akbari A., Noorbakhsh N., Moghadam N.B., Mamoudifard M. (2021). A Comprehensive Review on Efficient Approaches for Combating Coronaviruses. Biomed. Pharmacother..

[69] Malik J.A., Ahmed S., Mir A., Shinde M., Bender O., Alshammari F., Ansari M., Anwar S. (2022). The SARS-CoV-2 Mutations versus Vaccine Effectiveness: New Opportunities to New Challenges. J. Infect. Public Health.

[70] Fiolet T., Kherabi Y., MacDonald C.-J., Ghosn J., Peiffer-Smadja N. (2022). Comparing COVID-19 Vaccines for Their Characteristics, Efficacy and Effectiveness against SARS-CoV-2 and Variants of Concern: A Narrative Review. Clin. Microbiol. Infect..

[71] Andrews N., Tessier E., Stowe J., Gower C., Kirsebom F., Simmons R., Gallagher E., Thelwall S., Groves N., Dabrera G. (2022). Duration of Protection against Mild and Severe Disease by Covid-19 Vaccines. N. Engl. J. Med..

[72] Cascella M., Rajnik M., Aleem A. (2022). Features, Evaluation, and Treatment of Coronavirus (COVID-19).

[73] Forni G., Mantovani A., Forni G., Mantovani A., Moretta L., Rappuoli R., Rezza G., Bagnasco A., Barsacchi G., Bussolati G. (2021). COVID-19 Vaccines: Where We Stand and Challenges Ahead. Cell Death Differ..

[74] Bezbaruah R., Borah P., Kakoti B.B., Al-Shar’I N.A., Chandrasekaran B., Jaradat D.M.M., Al-Zeer M.A., Abu-Romman S. (2021). Developmental Landscape of Potential Vaccine Candidates Based on Viral Vector for Prophylaxis of COVID-19. Front. Mol. Biosci..

[75] Robert-Guroff M. (2007). Replicating and Non-Replicating Viral Vectors for Vaccine Development. Curr. Opin. Biotechnol..

[76] Callaway E. (2020). The Race for Coronavirus Vaccines: A Graphical Guide. Nature.

[77] Sadoff J., Gray G., Vandebosch A., Cárdenas V., Shukarev G., Grinsztejn B., Goepfert P.A., Truyers C., Fennema H., Spiessens B. (2021). Safety and Efficacy of Single-Dose Ad26.COV2.S Vaccine against Covid-19. N. Engl. J. Med..

[78] Alter G., Yu J., Liu J., Chandrashekar A., Borducchi E.N., Tostanoski L.H., McMahan K., Jacob-Dolan C., Martinez D.R., Chang A. (2021). Immunogenicity of Ad26.COV2.S Vaccine against SARS-CoV-2 Variants in Humans. Nature.

[79] WHO (2021). The Janssen Ad26.COV2.S COVID-19 Vaccine: What You Need to Know.

[80] Watanabe Y., Mendonça L., Allen E.R., Howe A., Lee M., Allen J.D., Chawla H., Pulido D., Donnellan F., Davies H. (2021). Native-like SARS-CoV-2 Spike Glycoprotein Expressed by ChAdOx1 NCoV-19/AZD1222 Vaccine. ACS Cent. Sci..

[81] Dicks M.D.J., Spencer A.J., Edwards N.J., Wadell G., Bojang K., Gilbert S.C., Hill A.V.S., Cottingham M.G. (2012). A Novel Chimpanzee Adenovirus Vector with Low Human Seroprevalence: Improved Systems for Vector Derivation and Comparative Immunogenicity. PLoS ONE.

[82] World Health Organization (WHO) (2022). The Oxford/AstraZeneca (ChAdOx1-S [Recombinant] Vaccine) COVID-19 Vaccine: What You Need to Know.

[83] Vanaparthy R., Mohan G., Vasireddy D., Medicina P.A.-I. (2021). Review of COVID-19 Viral Vector-Based Vaccines and COVID-19 Variants. Infez. Med..

[84] Klassen S.A., Senefeld J.W., Senese K.A., Johnson P.W., Wiggins C.C., Baker S.E., van Helmond N., Bruno K.A., Pirofski L.A., Shoham S. (2021). Convalescent Plasma Therapy for COVID-19: A Graphical Mosaic of the Worldwide Evidence. Front. Med..

[85] Hindustan Times. All You Need to Know about SII Vaccine ‘Covishield’|10 Points|Latest News India Hindustan Times. https://www.hindustantimes.com/india-news/ingredients-side-effects-response-time-all-you-need-to-know-about-covishield-101610469717522.html.

[86] Business Standard. Rejection of Sputnik V by US, EU a Mistake: Argentine Epidemiologist. Business Standard News. https://www.business-standard.com/article/international/rejection-of-sputnik-v-by-us-eu-a-mistake-argentine-epidemiologist-121122800647_1.html.

[87] Chavda V.P., Patel A.B., Vaghasiya D.D. (2022). SARS-CoV-2 variants and vulnerability at the global level. J. Med. Virol..

[88] PHO (2021). COVID-19 Vaccines: Viral Vector-Based Vaccines The Basics: Viral Vector-Based Vaccines.

[89] Kyriakidis N.C., López-Cortés A., González E.V., Grimaldos A.B., Prado E.O. (2021). SARS-CoV-2 Vaccines Strategies: A Comprehensive Review of Phase 3 Candidates. NPJ Vaccines.

[90] Awate S., Babiuk L.A., Mutwiri G. (2013). Mechanisms of Action of Adjuvants. Front. Immunol..

[91] Pulendran B., Arunachalam P.S., O’Hagan D.T. (2021). Emerging Concepts in the Science of Vaccine Adjuvants. Nat. Rev. Drug Discov..

[92] van Riel D., de Wit E. (2020). Next-Generation Vaccine Platforms for COVID-19. Nat. Mater..

[93] Mayo Clinic (2020). Different Types of COVID-19 Vaccines: How They Work.

[94] Coughlan L. (2020). Factors Which Contribute to the Immunogenicity of Non-Replicating Adenoviral Vectored Vaccines. Front. Immunol..

[95] Mendonça S.A., Lorincz R., Boucher P., Curiel D.T. (2021). Adenoviral Vector Vaccine Platforms in the SARS-CoV-2 Pandemic. NPJ Vaccines.

[96] Falsey A.R., Sobieszczyk M.E., Hirsch I., Sproule S., Robb M.L., Corey L., Neuzil K.M., Hahn W., Hunt J., Mulligan M.J. (2021). Phase 3 Safety and Efficacy of AZD1222 (ChAdOx1 NCoV-19) Covid-19 Vaccine. N. Engl. J. Med..

[97] Zheng C., Shao W., Chen X., Zhang B., Wang G., Zhang W. (2022). Real-world effectiveness of COVID-19 vaccines: A literature review and meta-analysis. Int. J. Infect. Dis..

[98] Funk C.D., Laferrière C., Ardakani A. (2020). A Snapshot of the Global Race for Vaccines Targeting SARS-CoV-2 and the COVID-19 Pandemic. Front. Pharmacol..

[99] World Health Organization (WHO) (2020). List of Candidate Vaccines Developed against SARS-CoV.

[100] McGill COVID19 Vaccine Tracker Team (2022). COVID-19 VACCINE TRACKER. https://covid19.trackvaccines.org/vaccines/.

[101] KBR (2021). Cellid’s Covid-19 Vaccine Candidate Shows No Difference between Middle, High Doses.

[102] U.S. National Library of Medicine (2021). Evaluate the Safety, Immunogenicity and Potential Efficacy of an RVSV-SARS-CoV-2-S Vaccine.

[103] U.S. National Library of Medicine (2021). Phase 2b/3 Trial of VSV-ΔG SARS-CoV-2 Vaccine (BRILIFE) Against Approved Comparator Vaccine.

[104] Chinese Clinical Trail Registry (2021). A Phase I Clinical Trial of Influenza Virus Vector COVID-19 Vaccine for Intranasal Spray (DelNS1-2019-NCoV-RBD-OPT1).

[105] U.S. National Library of Medicine (2021). A Phase II Clinical Trial of Influenza Virus Vector COVID-19 Vaccine for Intranasal Spray (DelNS1-2019-NCoV-RBD-OPT1).

[106] U.S. National Library of Medicine (2021). A Phase III Clinical Trial of Influenza Virus Vector COVID-19 Vaccine for Intranasal Spray (DelNS1-2019-NCoV-RBD-OPT1).

[107] U.S. National Library of Medicine (2021). Safety and Immunogenicity Study of AdCLD-CoV19: A COVID-19 Preventive Vaccine in Healthy Volunteers.

[108] U.S. National Library of Medicine (2021). Preventive Dendritic Cell Vaccine, AV-COVID-19, in Subjects Not Actively Infected With COVID-19.

[109] U.S. National Library of Medicine (2021). Phase I–II Trial of Dendritic Cell Vaccine to Prevent COVID-19 in Adults.

[110] U.S. National Library of Medicine (2021). Dendritic Cell Vaccine to Prevent COVID-19.

[111] U.S. National Library of Medicine (2021). Dendritic Cell Vaccine, AV-COVID-19, to Prevent COVID-19 Infection.

[112] U.S. National Library of Medicine (2021). Safety and Immunogenicity of Two Different Strengths of the Inactivated COVID-19 Vaccine ERUCOV-VAC (ERUCOV-VAC).

[113] U.S. National Library of Medicine (2021). Efficacy, Immunogenicity and Safety of Inactivated ERUCOV-VAC Compared With Placebo in COVID-19.

[114] Pavel S.T.I., Yetiskin H., Uygut M.A., Aslan A.F., Aydın G., İnan Ö., Kaplan B., Ozdarendeli A. (2021). Development of an Inactivated Vaccine against SARS CoV-2. Vaccines.

[115] U.S. National Library of Medicine (2021). A Synthetic MVA-Based SARS-CoV-2 Vaccine, COH04S1, for the Prevention of COVID-19 Infection.

[116] U.S. National Library of Medicine (2021). SARS-CoV-2 Vaccine (COH04S1) Versus Emergency Use Authorization SARS-COV-2 Vaccine for the Treatment of COVID-19 in Patients With Blood Cancer.

[117] Rauch S., Jasny E., Schmidt K.E., Petsch B. (2018). New Vaccine Technologies to Combat Outbreak Situations. Front. Immunol..

[118] Eroglu B., Nuwarda R.F., Ramzan I., Kayser V. (2022). A Narrative Review of COVID-19 Vaccines. Vaccines.

[119] Kumar A., Kumar A. (2021). Mucosal and Transdermal Vaccine Delivery Strategies against COVID-19. Drug Deliv. Transl. Res..

[120] Shah D., Chavda V., Tandel H., Domadiya K. (2016). Nasal Medication Conveyance Framework: An Approach for Brain Delivery from Essential to Cutting Edge. Res. Rev. J. Med..

[121] U.S. National Library of Medicine (2021). A Phase I Clinical Trial of Influenza Virus Vector COVID-19 Vaccine for Intranasal Spray (DelNS1-2019-NCoV-RBD-OPT1).

[122] U.S. National Library of Medicine (2021). A Ph 2 Trial With an Oral Tableted COVID-19 Vaccine.

[123] Health Research Authority (2021). A First in Human Study of OraPro-COVID-19 in Healthy Volunteers.

[124] Abd Elkodous M., Olojede S.O., Morsi M., El-Sayyad G.S. (2021). Nanomaterial-Based Drug Delivery Systems as Promising Carriers for Patients with COVID-19. RSC Adv..

[125] Theobald N. (2020). Emerging Vaccine Delivery Systems for COVID-19 Functionalised Silica Nanoparticles Offer a Potentially Safe and Effective Alternative Delivery System for DNA/RNA Vaccines. Drug Discov. Today.

